# Crude Extracts from *Lycium barbarum* Suppress SREBP-1c Expression and Prevent Diet-Induced Fatty Liver through AMPK Activation

**DOI:** 10.1155/2014/196198

**Published:** 2014-06-10

**Authors:** Wang Li, Yan Li, Qing Wang, Yi Yang

**Affiliations:** ^1^Biochemistry and Molecular Biology, Ningxia Medical University, Yinchuan 750004, China; ^2^Key Laboratory of Fertility Preservation and Maintenance of Ministry of Education, Ningxia Medical University, Yinchuan 750004, China

## Abstract

*Lycium barbarum* polysaccharide (LBP) is well known in traditional Chinese herbal medicine that, has beneficial effects. Previous study reported that LBP reduced blood glucose and serum lipids. However, the underlying LBP-regulating mechanisms remain largely unknown. The main purpose of this study was to investigate whether LBP prevented fatty liver through activation of adenosine monophosphate-activated protein kinase (AMPK) and suppression of sterol regulatory element-binding protein-1c (SREBP-1c). Male C57BL/6J mice were fed a low-fat diet, high-fat diet, or 100 mg/kg LBP-treatment diet for 24 weeks. HepG2 cells were treated with LBP in the presence of palmitic acid. In our study, LBP can improve body compositions and lipid metabolic profiles in high-fat diet-fed mice. Oil Red O staining *in vivo* and *in vitro* showed that LBP significantly reduced hepatic intracellular triacylglycerol accumulation. H&E staining also showed that LBP can attenuate liver steatosis. Hepatic genes expression profiles demonstrated that LBP can activate the phosphorylation of AMPK, suppress nuclear expression of SREBP-1c, and decrease protein and mRNA expression of lipogenic genes *in vivo* or *in vitro*. Moreover, LBP significantly elevated uncoupling protein-1 (UCP1) and peroxisome proliferator-activated receptor-*γ* coactivator-1*α* (PGC-1*α*) expression of brown adipose tissue. In summary, LBP possesses a potential novel treatment in preventing diet-induced fatty liver.

## 1. Introduction

An imbalance between energy intake and expenditure can cause the excessive accumulation of triacylglycerol in tissues of eukaryotic organisms [1, 2]. The accumulation of triacylglycerol in adipose tissue can result in obesity and in nonadipose tissues, especially liver tissue, which are associated with type 2 diabetes and nonalcoholic fatty liver disease (NAFLD) [3, 4]. High-fat diet-induced fatty liver is characterized by excessive accumulation of triacylglycerol in the liver, also impairs fatty acid oxidation, and increases hepatic* de novo* lipogenesis [4–6]. Furthermore, long-term high-fat diet-induced dysregulation of hepatic lipid metabolism increases lipolysis, causing an increase in free fatty acid levels [7]. These metabolic changes trigger fatty liver and lead to systemic aggravation of lipid metabolic dysfunction [8].

Sterol regulatory element-binding protein-1c (SREBP-1c) is the key regulator of lipid metabolism on nutritional status. Activation of SREBP-1c increases hepatic lipogenesis under high dietary conditions and leads to fatty liver [9]. Conversely, activation of adenosine monophosphate-activated protein kinase (AMPK) has been shown to reduce lipogenesis in the liver by inhibiting SREBP-1c expression and further prevent the development of fatty liver [10]. Thus, some pharmacologic agents that inhibit SREBP-1c expression via stimulating AMPK activity in hepatocytes may provide more effective treatment options for fatty liver disease.

Previous studies have found that LBP was able to improve lipid metabolism profiles in animal and human models. For example, LBP clearly decreased serum total cholesterol (TC), triacylglycerol (TG), and high-density lipoprotein (HDL) in hyperlipidemic animal [11]. The clinical study provided evidence that LBP could play an important role in treating hyperlipidemic patients [12]. However, the underlying mechanisms of the hepatoprotective effects of LBP remain largely unknown. Our aim of this study is determine whether LBP has a protective effect against diet-induced fatty liver. To further explore and evaluate antilipid effects of LBP on the expression of SREBP-1c-mediated lipogenic genes involved in triacylglycerol synthesis through AMPK-dependent pathway were investigated in a model of diet-induced fatty liver* in vivo* and* in vitro*.

## 2. Material and Methods

### 2.1. Preparation of* Lycium barbarum* Polysaccharide (LBP)

LBP was extracted from* L. barbarum* as previously described [13]. Briefly, the dried fruit of* L. barbarum* was put in boiling deionized water. The water extract was filtered through a filter paper to remove impurities. The crude extract was concentrated to the volume under vacuum at 40°C and diluted to deionized water. Then the extract was precipitated with 95% ethanol, followed by centrifugation to remove the supernatant. Then the precipitate was collected and ground into powder. The powder of LBP was dissolved in normal saline for mice experiment, filtered through a 0.22 *μ*m filter, and stored at −20°C.

### 2.2. Animals and Diets

Male C57BL/6J mice from Beijing Vital River Biological Co., Ltd. were housed in standard cage conditions (temperature: 22 ± 1°C; relative humidity: 56 ± 5°C) at a 12/12 light/dark cycle. All mice had free access to diet and water for 2 weeks. Mice were divided into three groups (*n* = 10 per group): low-fat diet (LFD) (D12450B, USA), high-fat diet (HFD) (D12492, USA), and HFD plus LBP (100 mg/kg). Low-fat diet contains 10% of Kcal as fat with an energy density of 3.85 Kcal per gram, while high-fat diet contains 60% of Kcal as fat with an energy density of 5.24 Kcal per gram. We observed body weight, food intake, water intake, and blood glucose every week. We calculated food intake according to the following formula: food intake (g/d mouse) = the total food intake (g/per week)/[7 (d) × 5 (mice/cage)]. We calculated energy consumption according to the following formula provided by the research diets: energy consumption (Kcal/d mouse) = 3.85 or 5.24 (Kcal/g) × food intake (g/d mouse). All mice were deprived of diet and fasted for overnight at 24 weeks. Blood samples were collected from the eyeballs of mice and placed for 10 min at room temperature. Serum was obtained by centrifuging at 3,000 r for 15 min at 4°C and stored at −80°C. Liver and brown adipose tissue were isolated, frozen in liquid nitrogen, and stored at −80°C. The animal experiments were approved by the Animal Research Committee of Ningxia Medical University, China.

### 2.3. Cell Culture and Treatment

HepG2 cells (Peking University, China) were cultured in Dulbecco's modified Eagle's medium (DMEM) containing 30 mM glucose, 10% fetal bovine serum (Gibco, USA), and 1% penicillin and streptomycin (Invitrogen, CA, USA) in 5% CO_2_ at 37°C. The cells were pretreated with 250 *μ*M palmitate (Sigma) for 12 h and then treated with 30–600 *μ*g/mL LBP for 12 h.

### 2.4. Biochemical Analysis

The serum biochemical profiles, including TG, TC, AST, ALT, HDL, and LDL, were determined using a Biochem-Immunoautoanalyser (Brea, CA, USA). Glucose levels were determined with a glucometer (Accu-Chek; Roche Diagnostics). NEFA and DAG levels of the serum and liver tissue were measured with mouse ELISA kit (CUSABIO) according to the manufacturer's instructions. Triacylglycerol of liver tissue was measured with the triacylglyceride assay kit (Applygen Technologies Inc., China).

### 2.5. Histological Analysis

Liver tissue was fixed in 4% paraformaldehyde buffer, embedded in paraffin, and cut at 5 *μ*m sections. Liver sections were stained with hematoxylin and eosin (H&E). The assessment of liver histology was previously described [14]. To further confirm lipid droplet accumulation, seeded cells and frozen liver sections were stained with Oil Red O.

### 2.6. Immunohistochemistry

The sections were prepared as described for H&E staining. Immunohistochemistry was performed using the following primary antibodies: anti-rabbit-SREBP-1c and -FAS antibody and then stained with goat anti-rabbit IgG-HRP as secondary antibody. The reactivity of the antibodies was detected using a DAB horseradish peroxidase color development kit. The sections were counterstained with hematoxylin and observed with a microscope (Olympus IX71).

### 2.7. RNA Isolation and Semiquantitative and Quantitative RT- PCR

Total RNA was extracted from liver tissue using TRIzol reagent (Invitrogen). RNA concentrations were determined by SmartSpec Plus (BIO-RAD, USA). 1 *μ*g of total RNA was transcribed to cDNA using the superscript first-strand synthesis kit (Thermo) following instructions. UCP1 and PGC-1*α* expression were analyzed using semiquantitative RT-PCR. The cDNA was served as a template in a 50 *μ*L reaction mixture and was processed using an initial step at 94°C for 5 min, followed by 32 amplification cycles (94°C for 30 s, 55°C for 30 s, and 72°C for 35 s), and 72°C for 5 min. Primers were as follows: UCP1 (forward, 5′-GGTTTTGCACCACACTCCTG-3′; reverse, 5′-ACATGGACATCGCACAGCTT-3′), PGC-1*α* (forward, 5′-TAAATCTGCGGGATGATGGA-3′; reverse, 5′-GTTTCGTTCGACCTGCGTAA-3′), and GAPDH (forward, 5′-AGAACATCATCCCTGCATCC-3′; reverse, 5′-TCCACCACCCTGTTGCTGTA-3′). Quantitative RT-PCR analysis was performed with a LightCycler instrument (Roche Applied Science) and SYBR green detection of amplified products. Primers for SREBP-1c, ACC, FAS, CPT-1*α*, and CD36 were previously described [15]. PCR reactions were performed in triplicate and normalized to cyclophilin using the 2^−ΔΔCt^ method.

### 2.8. Western Blot

Whole and nuclear proteins were extracted from liver and HepG2 cells as described previously [10]. Protein concentration was quantified with the BCA method according to the supplier's instructions (Sigma). 50 *μ*g of protein was added to 8% SDS-PAGE and electroblotted onto PVDF membranes (Pall Corporation, Pensacola). The membranes were blocked and incubated with specific antibodies against AMPK, AMPK (pThr^172^), FAS, ACC (pSer^79^), and ACC (cell signaling technology) and SREBP-1c, *β*-actin, and Lamin A/C (Santa Cruz Biotechnology). Consequently, the membranes were incubated with the corresponding horseradish peroxidase goat anti-rabbit IgG-HRP or anti-mouse IgG-HRP as secondary antibody (Santa Cruz Biotechnology). The immunoreactive proteins were detected with enhanced chemiluminescence (Pierce Biotechnology, USA). Band intensities were scanned (Epson Perfection V33) and quantified using the Image J software.

### 2.9. Statistical Analysis

All results were expressed as means ± SEM. Data was analyzed using the ANOVA multiple comparison test (SPSS 13.0). *P* < 0.05 was considered to be statistically significant.

## 3. Results

### 3.1. Body Compositions Analysis

**Figure 1:**
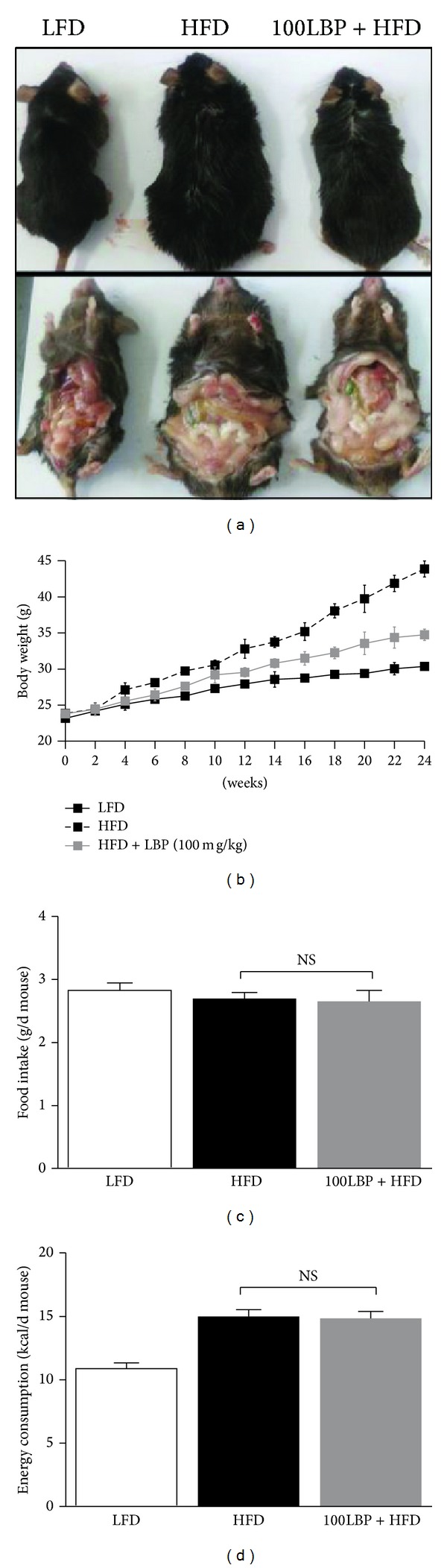
Effect of LBP on the whole statue of HFD-fed mice. (a) Gross contour of the whole body and visceral fat among four groups of mice for 24 weeks. (b) Changes in body weight every two weeks (*n* = 10 per group). ^#^ *P* < 0.05, compared to HFD group. (c) Food intake and (d) energy consumption. Data was expressed as mean ± SEM (*n* = 10 per group). NS: no significance. Note*:* food intake (g/d mouse) = the total food intake (g/per week)/[7 (d) × 5 (mice/cage)]; energy consumption (Kcal/d mouse) = 3.85 or 5.24 (Kcal/g) × food intake (g/d mouse).

**Figure 2:**
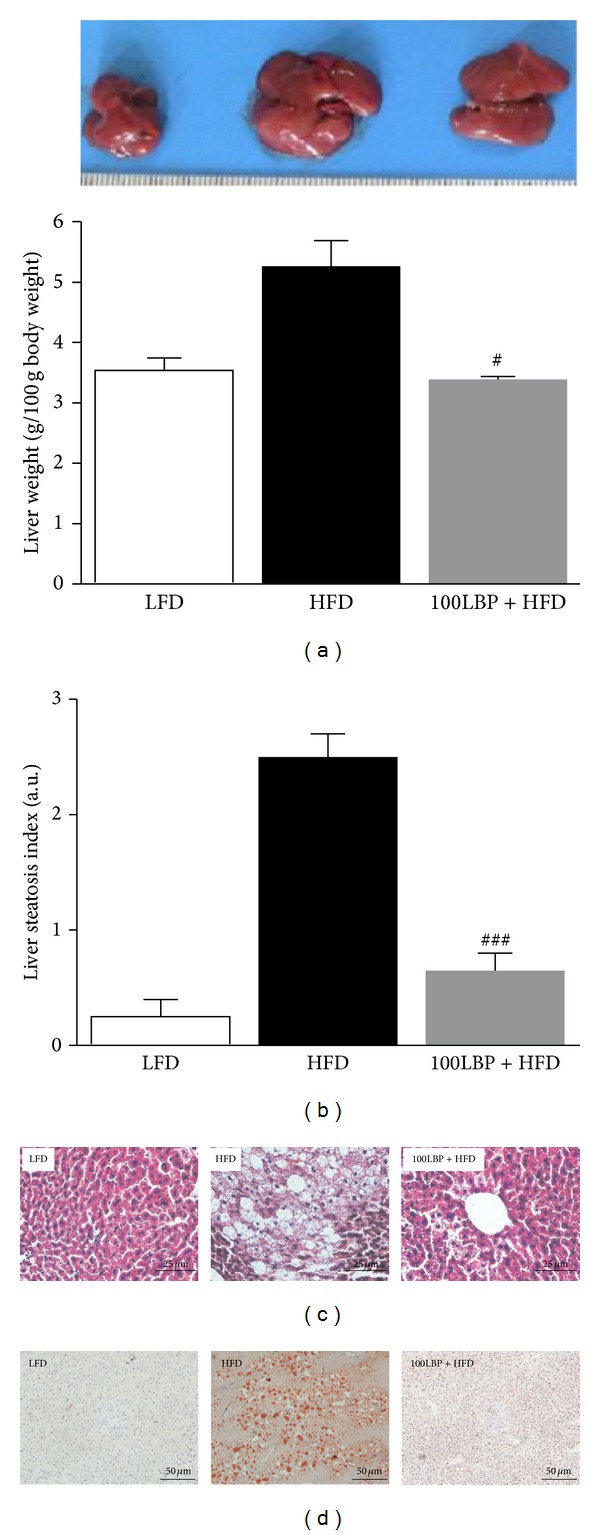
LBP reverses liver steatosis in HFD-fed mice. (a) Appearance of liver and percentage of hepatic mass. Data was expressed as mean ± SEM (*n* = 10 per group). ^#^ *P* < 0.05, compared to HFD group. (b) Liver steatosis index. ^###^ *P* < 0.001, compared to HFD group. (c) Representative photos of H&E-stained liver sections (×400 magnification). (d) Representative photos of Oil Red O-stained liver frozen sections (×200 magnification).

In the whole study, body weight, water intake, and food intake, which play a central role in HFD feeding mice, were monitored during the experiment. LBP significantly reduced body weight compared with HFD-fed mice (Figures 1(a) and 1(b) and Table 1, *P* < 0.05). There was no significance for food intake, energy consumption, and water intake between their groups (Figures 1(c) and 1(d) and Table 1). Abdominal circumference and BMI of LBP-treatment mice were significantly reduced as shown in Table 1 (*P* < 0.05). Liver weights and total white fat weights of HFD-fed mice were higher than the weights of LFD-fed mice and were reduced when mice were treated with LBP (Figure 2(a) and Table 1, *P* < 0.05 and *P* < 0.01). Moreover, LBP increased brown fat storages of HFD-fed mice (Table 1, *P* < 0.05). As shown in Figures 2(b) and 2(c), histological analysis of liver sections showed that LBP obviously lowered liver steatosis index compared to HFD-fed group (*P* < 0.001).

### 3.2. Serum Biochemistry Analysis

To further evaluate the effect of LBP-treatment mice, we measured serum TG, TC, HDL, LDL, AST, ALT, and NEFA. As shown in Table 2, serum concentrations of TC and LDL were all significantly lower in LBP-treatment mice than in HFD-fed mice, while HDL level was reversed in LBP-treatment mice (*P* < 0.05). LBP-treatment group had meaningful reduced NEFA levels compared to the HFD-fed group (*P* < 0.05). ALT and AST were also lower in LBP-treatment group than in HFD-fed group (*P* < 0.01). LBP also reduced blood glucose levels of the whole body of HFD-fed mice (*P* < 0.01). As shown in Table 3, we found that TG and DAG levels of serum and liver were significantly reduced in LBP-treatment group compared to that in HFD-fed group (*P* < 0.05). As expected, these data showed that treatment of LBP was valuable for improving the whole body composition and serum lipid profiles in HFD feeding mice.

### 3.3. LBP Activated the Phosphorylation of AMPK and Suppressed Nuclear SREBP-1c* In Vivo* and* In Vitro*

**Figure 3:**
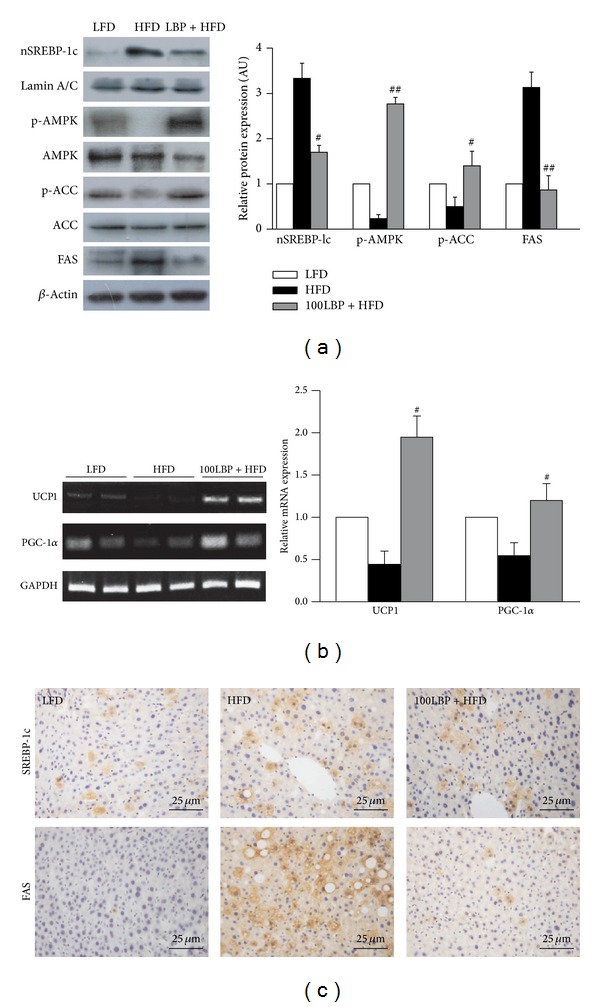
LBP prevents fatty liver by activation of AMPK and inhibition of SREBP-1c in HFD-fed mice. (a) Protein expression levels of p-AMPK, nuclear SREBP-1c, p-ACC, and FAS in liver tissue. Data was normalized to *β*-actin, ^#^ *P* < 0.05, and ^##^ *P* < 0.01, compared to HFD group, respectively. (b) The mRNA expression of UCP1 and PGC-1*α* in brown adipose tissue. Data was normalized to GAPDH and ^#^ *P* < 0.05, compared to HFD group, respectively. (c) Immunohistochemical analysis detected the distribution of SREBP-1c and FAS in liver sections (×400 magnification).

We assessed hepatic levels of phospho-AMPK, nuclear SREBP-1c, phospho-ACC, and FAS* in vivo* and* in vitro* experiments. Mice fed HFD had potently increased hepatic levels of nuclear SREBP-1c and FAS and decreased hepatic phosphorylation levels of AMPK and ACC. On the contrary, LBP significantly reversed HFD-induced alterations in hepatic levels of these proteins (Figures 3(a) and 5(a), *P* < 0.05 and *P* < 0.01). SREBP-1c and FAS staining showed cytoplasmic expression levels in liver, respectively (Figure 3(c)).

### 3.4. LBP Prevented Fatty Liver through AMPK/SREBP-1c Pathway

**Figure 4:**
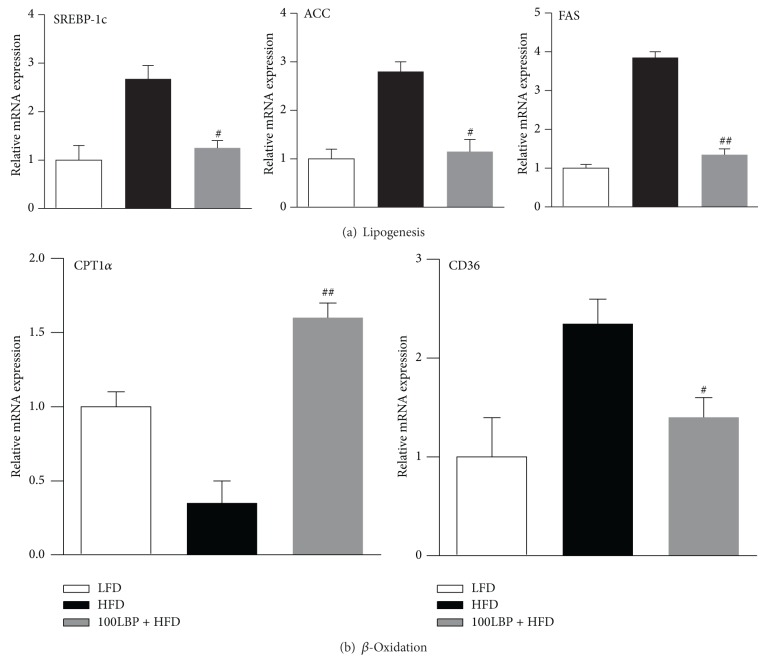
LBP improves the imbalance between lipogenesis and fatty acid *β*-oxidation via downregulation of SREBP-1c expression in HFD-fed mice. (a) Relative genes mRNA expression of lipogenesis and (b) fatty acid *β*-oxidation. Data was normalized to GAPDH, ^#^ *P* < 0.05, and ^##^ *P* < 0.01, compared to HFD group, respectively.

**Figure 5:**
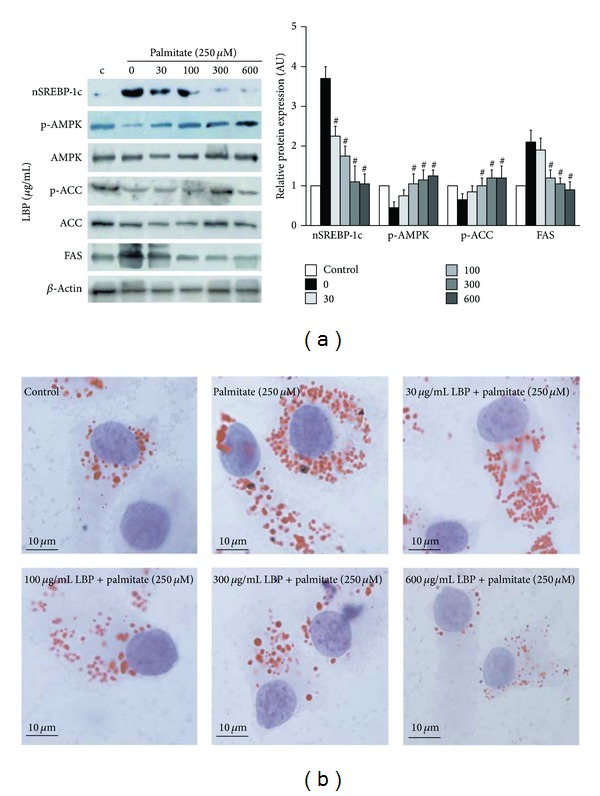
LBP reduces intracellular triacylglycerol accumulation via activation of AMPK and suppression of SREBP-1c in HepG2 cells. (a) HepG2 cells were pretreated with 250 *μ*M palmitate for 12 h and then treated with 30–600 *μ*g/mL LBP for 12 h. Western blot analyzed p-AMPK, nuclear SREBP-1c, p-ACC, and FAS expression. Data was normalized to *β*-actin and ^#^ *P* < 0.05, compared to HFD group, respectively. (b) Representative photos of Oil Red O-stained HepG2 cells (×1000 magnification).

We analyzed the mRNA levels of genes involved in lipogenesis and fatty acid **β*-oxidation*. Our results showed that mRNA levels of SREBP-1c, ACC, and FAS were increased in the HFD group compared to the LFD group. In contrast, LBP significantly reduced mRNA expression of these genes (Figure 4(a), *P* < 0.05 and *P* < 0.01). Interestingly, compared with mice fed HFD, LBP significantly elevated CPT-1*α* level and inhibited CD36 level (Figure 4(b), *P* < 0.05 and *P* < 0.01). Oil Red O staining* in vivo *and* in vitro* showed the same tendency in which LBP significantly reduced hepatic intracellular TG accumulation (Figures 2(d) and 5(b)). Taken together, LBP suppressed lipogenesis and stimulated fatty acid *β*-oxidation via AMPK/SREBP-1c pathway, reduced hepatic TG accumulation, and prevented fatty liver.

### 3.5. LBP Increased UCP1 and PGC-1*α* Expression of Brown Fat Tissue

We evaluated genes expression of UCP1 and PGC-1*α*. As shown in Figure 3(a), the mRNA levels of UCP1 and PGC-1*α* showed great differences between the three groups. The expressions of UCP1 and PGC-1*α* in HFD-fed mice were higher than that of ND-fed mice. LBP significantly enhanced these genes' expression of brown fat tissue in HFD-fed mice (*P* < 0.05).

## 4. Discussion

The mice model of high-fat diet-induced fatty liver was considered a good model for the study of metabolic syndrome [16, 17]. In our study, HFD-fed mice had obviously increased body and liver weights, serum concentrations of TG, DAG, TC, LDL, NEFA, AST, and ALT, and liver concentrations of TG and DAG. On the contrary, LBP significantly decreased body and liver weights as well as serum and liver lipid concentrations. LBP also attenuated the development of HFD-induced fatty liver as assessed by histochemical analysis. These data demonstrated that LBP possesses biological activities for the regulation of lipid metabolism and fatty liver development in the liver.

Although the molecular mechanism causing the development of fatty liver in the pathogenesis of nonalcoholic fatty liver disease (NAFLD) is complex, animal models have shown that modulating important enzymes, such as ACC and FAS, in fatty acid synthesis in liver may be a key for the treatment of NAFLD [18–20]. Recent studies in humans and in rodents have demonstrated that an increase in* de novo* hepatic lipogenesis plays a pivotal role in HFD-induced excessive hepatic fat accumulation [21, 22]. The* in vivo* and* in vitro* experiment showed overexpression of HFD-induced ACC and FAS was substantially suppressed by LBP. Hepatic expression of SREBP-1c, which plays a major role in fatty liver, is responsible for these genes in the liver [20, 23–25] and affects the lipid accumulation induced by high-fat diet [26, 27]. Our study showed that HFD-fed or palmitate-pretreatment mice stimulated hepatic expression of SREBP-1c at protein and mRNA levels* in vivo *and* in vitro*. However, SREBP-1c is regulated by multiple factors, including insulin [28], AMPK [10, 28, 29], and liver X receptors (LXR) [20, 29]. It has been shown that AMPK activators, including alpha-lipoic acid [30] and metformin [31], inhibit the SREBP-1c expression and prevent the development of fatty liver. Our results support such a mechanism, showing that suppression of SREBP-1c expression by LBP is mediated by stimulation of AMPK activity. Taken together, LBP regulates hepatic genes responsible for* de novo* fatty acid synthesis via modulation of AMPK/SREBP-1c pathway and diminishes HFD-induced fatty liver.

CPT-1*α* is a major enzyme that catalyzes fatty acid *β*-oxidation [32], while CD36 promotes a combination of lipid storage and lipogenesis [23, 33]. HFD-induced fatty liver further aggravated fatty acid *β*-oxidation system and impaired the balance of lipid metabolism [34]. Our study indicated that hepatic *β*-oxidation was indeed reduced in the HFD group. In contrast, treatment of LBP increased CPT-1*α* mRNA level and decreased CD36 mRNA level. These results suggested that the beneficial effects of LBP in the treatment of fatty liver may be partly due to improved fatty acid *β*-oxidation in the liver.

White adipose tissue (WAT) increases production and release of fatty acids and, in turn, leads to a fatty liver [35]. Unlike WAT, brown adipose tissue (BAT), an essential component of energy expenditure, expends energy by heat production through uncoupling protein-1 (UCP-1) and peroxisome proliferator–activated receptor-*γ* coactivator-1*α* (PGC-1*α*) in its mitochondria [36]. This change of HFD-induced downregulation of UCP-1 and PGC-1*α* in the brown adipocytes causes morphologic and metabolic conversion to the white adipocytes [35, 37, 38]. As expected, LBP significantly reversed the HFD-induced downregulation of UCP-1 and PGC-1*α* of brown adipose tissue. Therefore, it is possible that LBP activating or elevating the expression of UCP-1 and PGC-1*α* would have crucial effect on increasing the energy expenditure to ultimately prevent fatty liver, which in turn protects liver function.

## 5. Conclusion

In conclusion, our present results, for the first time, demonstrate that LBP ameliorates HFD-induced fatty liver* in vivo* and* in vitro*, in which modulation of the hepatic AMPK/SREBP-1c pathway plays a pivotal role. Our finding may provide better understanding of LBP and associated chemicals and herbs in the treatment of liver disorders, especially nonalcoholic fatty liver disease.

## Figures and Tables

**Table 1:** Effect of LBP on body compositions of HFD-fed mice.

Variables	LFD	HFD	100 LBP + HFD
Initial body weight (g)	23.1 ± 0.23	23.8 ± 0.29	23.6 ± 0.23
Final body weight (g)	30.0 ± 0.46	44.6 ± 1.01	33.5 ± 1.43^#^
Water intake (mL/d/mouse)	6.97 ± 0.16	8.41 ± 0.17	7.26 ± 0.05
Abdominal circumference (cm)	7.63 ± 0.13	10.83 ± 0.08	7.98 ± 0.14^#^
BMI (g/cm^2^)	0.32 ± 0.07	0.58 ± 0.10	0.37 ± 0.09^#^
Liver weight (g)	1.06 ± 0.04	2.31 ± 0.18	1.25 ± 0.05^#^
White fat weight (g)	0.71 ± 0.06	4.40 ± 0.22	1.66 ± 0.08^##^
Brown fat weight (g)	0.24 ± 0.03	0.09 ± 0.01	0.30 ± 0.05^##^

Data are expressed as means ± SEM (*n* = 10 per group), ^#^ *P* < 0.05 and ^##^ *P* < 0.01, compared to HFD group. LFD: low-fat diet; HFD: high-fat diet; 100 LBP + HFD: 100 mg/kg LBP plus high-fat diet; BMI: body mass index.

**Table 2:** Effect of LBP on serum lipid profile of HFD-fed mice.

Variables	LFD	HFD	100 LBP + HFD
Blood glucose (mmol/L)	3.82 ± 0.15	9.66 ± 0.19	5.02 ± 0.26^##^
Total cholesterol (mmol/L)	1.24 ± 0.56	2.09 ± 1.14	1.38 ± 2.04^#^
HDL-cholesterol (mmol/L)	0.41 ± 0.03	0.28 ± 0.03	0.58 ± 0.09^#^
LDL-cholesterol (mmol/L)	0.13 ± 0.12	0.55 ± 0.25	0.28 ± 0.46^#^
AST (U/L)	78.4 ± 8.70	244.6 ± 12.45	141.7 ± 5.26^##^
ALT (U/L)	68.6 ± 3.83	129.3 ± 6.36	78.2 ± 5.18^##^
NEFA (umol/L)	244 ± 10.8	480 ± 26.5	261 ± 33.6^###^

Data are expressed as means ± SEM (*n* = 10 per group), ^#^ *P* < 0.05, ^##^ *P* < 0.01, and ^###^ *P* < 0.001, compared to HFD group. HDL: high-density lipoprotein; LDL: low-density lipoprotein; AST: aspartate aminotransferase; ALT: alanine aminotransferase; NEFA: nonesterified fatty acids.

**Table 3:** Effect of LBP on DAG and TG levels of serum and liver of HFD-fed mice.

Variables		LFD	HFD	100 LBP + HFD
DAG	Serum (pmol/L)	3.16 ± 0.08	6.27 ± 0.50	3.50 ± 0.34^#^
	Liver (nmol/g)	0.59 ± 0.09	1.03 ± 0.03	0.57 ± 0.07^#^

TG	Serum (mmol/L)	0.84 ± 0.16	1.83 ± 0.27	1.02 ± 0.48^#^
	Liver (mg/g)	2.2 ± 0.56	5.6 ± 0.97	2.8 ± 0.55^#^

Data are expressed as mean ± SEM (*n* = 10 per group), ^#^ *P* < 0.05, compared to HFD group. DAG: diacylglycerol; TG: triacylglycerol.

## Abbreviations

ACC: Acetyl-CoA carboxylase
CD36: Cluster of differentiation 36
CPT-1*α*: Carnitine palmitoyltransferase 1*α*
DAG: Diacylglycerol
FAS: Fatty acid synthetase
HFD: High-fat diet
LBP: * Lycium barbarum* polysaccharide
NAFLD: Nonalcoholic fatty liver disease
LFD: Low-fat diet
NEFA: Nonesterified fatty acid
PGC-1*α*: Peroxisome proliferator-activated receptor-*γ* coactivator-1*α*
SREBP-1c: Sterol regulatory element-binding protein-1c
UCP1: Uncoupling protein-1.
